# The impact of COVID-19 pandemic on cardiac surgery in Israel

**DOI:** 10.1186/s13019-020-01342-5

**Published:** 2020-10-02

**Authors:** Eitan Keizman, Eilon Ram, Erez Kachel, Leonid Sternik, Ehud Raanani

**Affiliations:** 1 Department of Cardia cSurgery, The Leviev Cardiothoracic and Vascular Center, Sheba Medical Center, Affiliated to the Sackler School of Medicine, Tel Aviv University, Ramat-Gan, Israel; 2 Department of Cardiac Surgery, The Baruch Padeh Medical Center, 52621 Tiberias, Israel

**Keywords:** Cardiac surgery, COVID-19

## Abstract

**Background:**

Ever since the coronavirus disease 2019 (COVID-19) has become a pandemic, worldwide efforts are being made to “flatten the curve”. Israel was amongst the first countries to impose significant restrictions. As a result, cardiac surgeons have been required to scale down their routine practice, resulting in a significant reduction in the number of cardiac surgeries. The aim of this study is to characterize the impact of COVID-19 on cardiac surgery in Israel.

**Methods:**

This is a retrospective observational study performed in two cardiac surgery departments in Israel and includes all patients who underwent cardiac surgery in March and April during the years 2019 and 2020. The patient cohort was divided into two groups based on the year of operation. Analysis of the patients’ baseline characteristics, operative data, and postoperative outcome, was performed.

**Results:**

The 2019 group (*n* = 173), and the 2020 group (*n* = 108) were similar regarding their baseline characteristics, previous medical history, and rates of previous revascularization interventions. However, compared to the 2019 group, patients in the 2020 group were found to be more symptomatic (NYHA class IV; 2.4% vs. 6.2%, *p* = 0.007). While all patients underwent similar procedures, patients in the 2020 group had significantly longer procedural time (*p* < 0.001). In-hospital mortality rate was found to be significantly higher in group 2020 (13% vs. 5.2%, *p* = 0.037).

**Conclusions:**

While the number of patients undergoing cardiac surgery declined during the outbreak period, the rate of surgical mortality increased. One explanation for this might be delayed hospital arrival.

## Introduction

Towards the end of 2019 a new disease emerged in Wuhan, China, now known as coronavirus disease 2019 (COVID-19), caused by severe acute respiratory syndrome coronavirus 2 (SARS-CoV-2) [1–3]. Approximately 3 months later, on March 11, 2020, COVID-19 was announced by the World Health Organization as a global pandemic. As of May 20, 2020, more than five million people worldwide were affected by the virus with about 325,000 deaths. Since the beginning of COVID-19 the world has changed drastically, and various measures have been taken in an attempt to “flatten the curve”, some of them having an unprecedented effect on world health systems [4].

Israel was amongst the first countries to close its borders and impose significant restrictions on its population, comprising personal movement restrictions, social distancing and cancellation of public transportation. In early March, schools and stores were closed, and dedicated units to treat COVID-19 patients were inaugurated. By the time COVID-19 was declared a pandemic, it had affected approximately 200 people countrywide with no fatalities.

Being a small country, one of the primary goals of the system was to anticipate the influx of patients arriving at medical centers in order to prevent a collapse of medical resources, including exhaustion of personnel, lack of equipment, protective gear, mechanical ventilators, surgical drapes, and extracorporeal membrane oxygenation (ECMO) machines. Virtually all medical specialties were required to significantly reduce their daily routine services. Specifically, cardiac surgeons were requested to scale down their clinical practice and adapt to a new hospital policy [4–6]. As a result, cardiac surgical programs engaged into a new reality of patient selection, prioritizing urgent cases while delaying elective procedures. Similar circumstances are reported worldwide [1, 4, 5, 7]. Additionally, from fear of getting infected, a new phenomenon of avoiding medical care emerged, and thus, non-COVID-19 patients requiring cardiac surgery refrained from requesting help.

While the number of patients undergoing cardiac surgery compared with previous years has undoubtedly decreased, the actual impact of this decline is yet to be fully understood. Therefore, the aim of this study is to characterize the impact of COVID-19 on cardiac surgery discipline by comparing the same time periods from different years within two major cardiac surgery departments in Israel.

## Methods

This is a retrospective observational study performed in two cardiac surgery departments in Israel: the Leviev Heart Center, Sheba Medical Center, Tel Hashomer, the largest tertiary hospital in Israel, located in the center of the country, and the Baruch Padeh Medical Center, Poriya, which is located in the north of the country.

All patients undergoing cardiac surgery between March 1 and April 30 during the years 2019 and 2020 were included in the cohort. No exclusion criteria applied. The patients were divided into two groups based on the year in which they underwent surgery. The decision to investigate specifically March and April was based on the fact that the strict limitations came into force at the beginning of March and continued throughout those 2 months.

A careful analysis of the patients’ baseline characteristics, operative data, and postoperative outcomes, was performed by two separate researchers. The data reviewed included hospitalization records, outpatient clinic visits, and the records of local and referring physicians. Throughout the study period, all procedures were performed and completed by the same surgical and anesthesiology teams.

### Statistical analysis

Data are presented as mean ± standard deviation. Continuous variables were tested with the Kolmogorov-Smirnov test for normal distribution. Categorical variables are given as frequencies and percentages. A chi-square test was used for comparison of categorical variables between the groups. Student’s t-test was performed for comparison of normally distributed continuous variables and Mann-Whitney U test for non-normal distribution. Statistical significance was assumed when the null hypothesis could be rejected at *P* < 0.05. All *P*-values are the results of two-sided tests. Statistical analyses were conducted using R (version 3.4.1).

## Results

### Baseline characteristics and operative data

The 2019 group included 173 patients, while group 2020 included 108 patients, accounting for a reduction in cases of more than a third from the previous year (37.5%). Baseline characteristics of patients in both groups were similar. The mean age was 64 ± 14 years and 70% were men. Furthermore, both groups were similar in terms of previous medical history and pre-existing conditions including hypertension, dyslipidemia, diabetes mellitus, insulin dependence, chronic renal failure, dialysis therapy, peripheral vascular disease, chronic obstructive pulmonary disease, pulmonary hypertension, prior cerebral event, previous cardiac surgery and atrial fibrillation (Table 1). Both groups had similar rates of previous revascularization interventions including both percutaneous cardiac intervention (PCI) and coronary artery bypass graft (CABG). However, there was a trend toward a higher EuroSCORE in the 2020 compared to the 2019 group (Table 1). Additionally, compared to the 2019 group, patients in the 2020 group were found to have more severe symptoms, reflected by higher New York Heart Association (NYHA) functional class IV (2.4% vs. 6.2%, *p* = 0.007).

**Table 1:** Baseline characteristics

	2019 Group *N* = 173	2020 Group *N* = 108	*p*-value
Age (mean ± SD)	63.5 ± 13.5	64 ± 13.7	0.765
Gender (male) (%)	128 ± 74	69 ± 63.9	0.096
Height (mean ± SD)	168.9 ± 17.3	166.4 ± 16.2	0.251
Weight (mean ± SD)	78.6 ± 16.5	80.4 ± 21	0.440
BMI (mean ± SD)	41.8 ± 183.5	31.4 ± 25.9	0.592
Obesity (%)	91 (53)	54 (61)	0.287
Hypertension (%)	112 (66)	61 (71)	0.501
PVD (%)	14 (8)	5 (8.2)	1.000
Diabetes (%)	68 (39)	40 (46)	0.370
Insulin treatment (%)	2 (7)	1 (7.7)	1.000
Hyperlipidemia (%)	109 (65)	57 (66)	0.984
Atrial fibrillation (%)	102 (59)	52 (58)	1.000
Previous MI (%)	18 (58)	10 (77)	0.399
Previous revascularization (%)			0.087
None	28 (90)	9 (69)	
PCI	2 (7)	4 (31)	
CABG	1 (3)	0 (0)	
Renal impairment (%)	12 (7)	12 (14)	0.126
Baseline creatinine (mean ± SD)	1.05 ± 0.63	1.2 ± 0.95	0.160
COPD (%)	14 (8)	11 (14)	0.272
Prior stroke(%)	14 (8)	3 (3)	0.223
Dialysis (%)	1 (0.6)	2 (2.3)	0.552
Previous cardiac operation (%)	3 (10)	1 (8)	1.000
Pulmonary hypertension (> 60 mmHg) (%)	0 (0)	2 (15)	0.149
LVEF (%) (mean ± SD)	52 ± 13.4	52.2 ± 13.6	0.903
NYHA functional class IV (%)	4 (2)	6 (6)	0.007
EuroSCORE (standard) (mean ± SD)	4.89 ± 2.78	5.73 ± 3.56	0.086
EuroSCORE (logistic) (mean ± SD)	5.41 ± 7.68	7.78 ± 11.47	0.098

*SD* Standard deviation, *BMI* Body mass index, *PVD* Peripheral vascular disease, *MI* Myocardial infarction, *PCI* Percutaneous coronary intervention, *CABG* Coronary artery bypass grafting, *COPD* Chronic obstructive pulmonary disease, *LVEF* Left ventricle ejection fraction, *NYHA* New York Heart Association

While patients from both years underwent similar surgical procedures, patients in the 2020 group had significantly longer cross-clamp time (68 ± 30 vs. 107 ± 88 min, *p* = 0.005) (Table 2).

**Table 2:** Surgical procedures

	2019 Group *N* = 173	2020 Group *N* = 108	*p*-value
AVR (%)	45 (26.0)	24 (22.2)	0.565
MVR/MVr (%)	31 (17.9)	25 (23.1)	0.361
TVr (%)	13 (7.5)	4 (3.7)	0.295
Ascending aorta repair (%)	17 (9.8)	6 (5.6)	0.295
Dissection repair (%)	4 (2.3)	2 (1.9)	1.000
MAZE procedure (%)	4 (2.3)	6 (5.6)	0.273
CABG (%)	87 (50.3)	54 (50.0)	1.000
Number of total grafts (%)			0.217
1	18 (24.0)	8 (15.4)	
2	29 (38.7)	15 (28.8)	
3	21 (28.0)	23 (44.2)	
4	7 (9.3)	6 (11.5)	
Cross clamp time, minutes (%)	68.4 (30.5)	107.4 (87.6)	0.005

*AVR* Aortic valve replacement, *MVR* Mitral valve replacement, *MVr* Mitral valve repair, *TVr* Tricuspid valve repair, *CABG* coronary artery bypass graft

### Postoperative outcomes

The major finding was the in-hospital mortality rate, which was found to be significantly higher in group 2020, with 14 (13.0%) deaths versus 9 (5.2%) deaths in 2019 (*p* = 0.037). While there was no emergency reopening for bleeding or tamponade in 2020, 8 patients required surgical revision in 2019 (4.7%) (*p* = 0.058). There were no significant differences between the groups regarding other postoperative complications, which included: stroke, acute respiratory distress syndrome (ARDS), heart failure, acute kidney injury, atrial fibrillation, permanent pacemaker implantation, and wound infection (Table 3). All patients in the 2020 cohort tested negative for COVID-19 during hospitalization prior to operation.

**Table 3:** Postoperative Outcomes

	2019 Group *N* = 173	2020 Group *N* = 108	*p*-value
In-hospital mortality (%)	9 (5.2)	14 (13.0)	0.037
Revision (%)	8 (4.7)	0 (0.0)	0.058
Stroke (%)	0 (0)	0 (0)	1.000
AKI (%)			0.233
Up to: mild	170 (98.8)	104 (97.2)	
moderate	1 (0.6)	3 (2.8)	
severe	1 (0.6)	0 (0.0)	
ARDS (%)	1 (3.3)	2 (16.7)	0.394
AF (%)	48 (27.9)	20 (22.2)	0.396
PPS (%)	20 (11.6)	13 (14.4)	0.648
HF (%)	1 (3.3)	1 (8.3)	1.000
Pacemaker (%)	1 (3.3)	0 (0.0)	1.000
Wound infection (%)			0.242
None	164 (95.3)	87 (96.7)	
Superficial	8 (4.7)	2 (2.2)	
Deep	0 (0.0)	1 (1.1)	
Ventilation time, minutes (%)	2721.28 (7779.54)	4070.48 (9294.47)	0.231
ICU time, hours (%)	71.43 (160.64)	95.27 (180.40)	0.298
Hospital time, days (%)	9.95 (8.39)	9.84 (6.82)	0.916

*AKI* acute kidney injury, *ARDS* acute respiratory distress syndrome, *AF* atrial fibrillation, *PPS* Peripheral pulmonic stenosis, *HF* heart failure, *ICU* intensive care unit

## Discussion

The developed world is currently facing what seems to be a watershed, and no doubt one of the significant landmarks in the history of medicine. One of the greatest challenges of the medical community is the scarcity of knowledge and experience in facing this virus [2, 3].

Apart from the common typical viral symptoms (i.e. fever, cough, fatigue, dyspnea, and diarrhea) or, in more severe cases, pneumonia and ARDS, COVID-19 also has some significant consequences on the cardiovascular system and the management of cardiovascular patients. The first association between COVID-19 and the cardiovascular system is the increased risk in patients with pre-existing cardiovascular disease to develop severe disease and death. Second, complications such as myocarditis [8–10] acute myocardial infarction [11], arrhythmias [9], thromboembolic events [12–14], and heart failure [11, 14], have been linked to COVID-19 infection. In some cases, these complications engendered treatment with ECMO [15–17]. Third, reports on cardiovascular side effects of COVID-19 therapies have been published [18–20], and lastly, the pandemic’s consequences regarding non-COVID-19 cardiovascular patients. New policies have affected virtually each and every medical discipline, specifically those of surgeons in various fields who were required to apply a triage never before seen in their daily routine practice [3, 6, 7]. Meanwhile, increasing numbers of patients are avoiding medical care while being symptomatic at home. As the pandemic is still ongoing, the aftermath remains largely unclear, with emerging reports of patients deteriorating or dying at home. However, it will be difficult to quantify the impact of non-COVID-19 morbidity and mortality during the COVID-19 pandemic. While the number of infected patients is objectively quantifiable, the number of non-COVID-19 patients suffering from the outbreak’s consequences is far from being measurable. In other words, the impact of the virus is not solely from personally transmitted infection.

One of the aspects of this may be illustrated in the field of cardiac surgery. At the beginning of the outbreak, Légaré*et al*., on behalf of the Canadian Society of Cardiac Surgeons (CSCS), released a guidance statement to cardiac surgeons. They suggested a template for triaging patients based on the percentage in reduction of services. According to their suggestion, upon a mild reduction in services (0–30%), only symptomatic outpatients or those at greater risk for developing adverse events, should undergo surgery alongside the urgent cases. Under a > 50% reduction in services, they suggested operating on urgent cases only [4, 7].

Israel was one of the first responders to the crisis, and consequently there has been a significant reduction in the number of cardiac surgeries performed. Evidently, characterizing the preoperative status of the entire patient cohort, those of the COVID-19 era were not necessarily sicker, but rather more symptomatic. Coincidentally or not, this correlates with the CSCS recommendation, although no guidance statement has been released by the Israel Society of Cardiothoracic Surgery. Apart from the obvious reasons, another consideration advocating for the delay of asymptomatic elective cases is the incubation period of COVID-19. A recent paper reported on patients who tested negative for COVID-19 during their asymptomatic incubation period and then underwent various surgical procedures. The mortality rate of these patients was dramatically high (20.5%) [21]. Conversely, it could be argued that symptoms are subjective rather than objective parameters, and there might be some clinical discrepancies between NYHA and the severity of the disease.

One of the most intriguing facts demonstrated by this study emerged from the operative data. While there was no net difference in terms of types and complexity of procedures, patients from the COVID-19 era had longer procedural time. It could be that the cases selected in 2020 were more complex and challenging. However, it is difficult to ascertain whether the underlying reason for this is a more complex anatomy, late hospital arrival, or simply a coincidence.

Several factors may contribute to the higher rate of in-hospital mortality for patients in the 2020 group (13% vs. 5.2%). The first is the aforementioned assumption of more challenging procedures. The second hypothesis is possibly related to late hospital arrival of patients during the COVID-19 pandemic, which may have worsened their underlying condition. With the pandemic progressing worldwide, panic grew within the population, and indeed patients avoided hospitals even at the cost of being symptomatic.

Lastly, cardiopulmonary bypass might have a deleterious effect on COVID-19 patients undergoing cardiac surgery. This is explained by an increased inflammatory response triggered by the non-endothelial surfaces of the pump [22]. However, as off-pump CABG is not routinely performed in our institute, this study was unable to evaluate a comparison between this possible effect of on-pump CABG.

May 2020 was a positive turning point in Israel concerning COVID-19. The “curve” seemed to be flattened and the number of new positive cases declined daily, together with the governmental restrictions including those concerning medical practices. This prompted the question of when to restore the daily routine in the field of interventional cardiology and cardiac surgery. In fact, the number of patients is growing on a daily basis and elective cases are now performed routinely. The safety of the medical teams remains the first priority, and the primary goal is to proactively manage all surgical cases including comprehensive COVID-19 screening protocol for both patients and personnel.

Now that the first major outbreak of COVID-19 seems to be fading away, cardiac surgeons must bear in mind that a second outbreak might be coming soon. Therefore, it is imperative that cardiac surgery teams should be vigilant and learn from their own experience and the experience of others. It is a call for surgeons to be aware of the possible and probable impact of COVID-19 on non-COVID-19 patients.

## Conclusion

COVID-19 is having an impact on both infected and non-infected patients. During the outbreak period, a smaller number of patients underwent cardiac surgery. These patients experienced more symptoms, had longer procedures, and had a higher in-hospital mortality rate. While the consequences of the pandemic are not fully understood, one thing is certain; we must prepare for a possible new outbreak.

## Data Availability

Supporting data are available.

## Abbreviations

ARDS: acute respiratory distress syndrome
CABG: coronary artery bypass graft
COVID-19: coronavirus disease 2019
CSCS: Canadian Society of Cardiac Surgeons
ECMO: extracorporeal membrane oxygenation
NYHA: New York Heart Association
PCI: percutaneous cardiac intervention
SARS-CoV-2: severe acute respiratory syndrome coronavirus 2
